# Comparing the salinity tolerance of twenty different wheat genotypes on the basis of their physiological and biochemical parameters under NaCl stress

**DOI:** 10.1371/journal.pone.0282606

**Published:** 2023-03-31

**Authors:** Amaneh Ghasemi Masarmi, M Solouki, B Fakheri, Hazem M. Kalaji, N Mahgdingad, S Golkari, Arkadiusz Telesiński, Sobhi F. Lamlom, Henryk Kociel, Ahmed Fathy Yousef

**Affiliations:** 1 Plant Breeding- Genetic Biometric, University of Zabol, Zabol, Iran; 2 Faculty of Agriculture, Department of Plant Breeding and Biotechnology, University of Zabol, Zabol, Iran; 3 Department of Plant Physiology, Institute of Biology, Warsaw University of Life Sciences SGGW, Warsaw, Poland; 4 Institute of Technology and Life Sciences—National Research Institute, Raszyn, Poland; 5 Agriculture Research, Education and Extension Organization (AREEO), Dryland Agriculture Research Institute (DARI), Maragheh, Iran; 6 Department of Bioengineering, West Pomeranian University of Technology in Szczecin, Szczecin, Poland; 7 Faculty of Agriculture Saba Basha, Plant Production Department, Alexandria University, Alexandria, Egypt; 8 Firma Usługowa Ogród, Maria Kociel, Łuków, Poland; 9 Department of Horticulture, College of Agriculture, University of Al-Azhar (Branch Assiut), Assiut, Egypt; Government College University Lahore, PAKISTAN

## Abstract

The climate has drastically changed over the past two decades. Rising temperatures and climate change may lead to increased evapotranspiration, specifically soil evaporation, causing water to evaporate and salt to accumulate in the soil, resulting in increased soil salinity. As a result, there is a need to evaluate methods for predicting and monitoring the effects of salinity on crop growth and production through rapid screening. Our study was conducted on 20 wheat genotypes, 10 sensitive and 10 tolerant, exposed to two salinity levels (90 and 120 mM NaCl) with the control under greenhouse conditions. Our results revealed significant differences in the genotypes’ response to salinity. Salt stress decreased chlorophyll index in sensitive genotypes but increased chlorophyll *a* and carotenoids in tolerant genotypes at 90 mM. Salt stress also increased protein, proline, lipoxygenase, and reactive thiobarbituric acid levels in all wheat genotypes. The study suggests that plant photosynthetic efficiency is a reliable, non-destructive biomarker for determining the salt tolerance of wheat genotypes, while other biochemical traits are destructive and time-consuming and therefore not suitable for rapid screening.

## 1. Introduction

Wheat (*Triticum aestivum* L.) is an important staple food, providing nearly 30% of the world’s population (4.5 billion people) with calories and 20% of total protein requirements [1]. It is grown in many countries around the world to meet the food needs of the population. However, wheat yield per hectare is much lower than its capacity for several reasons, the most common being salinity, as shown by *Pramila*, *et al*. [2].

Salinity stress, which is caused by high salt levels in the soil, can negatively impact plant growth and development. It can lead to reduced water uptake, nutrient imbalances, and damage to the plant’s cells and tissues [3]. The detrimental effect of salinity stress basically occurs in two successive phases: (i) osmotic stress, and (ii) ionic toxicity (Na^+^ and Cl^−^), followed by the subsequent impact of secondary stresses such as oxidative stress and nutritional imbalances [4,5]. Low or moderate salinity mainly causes osmotic stress; it also affects the photosynthetic activity of the plant, resulting in low growth and yield [6]. It also weakens the light-collecting pigments known as photosynthetic pigments, which are present in the thylakoid membranes of chloroplasts and include carotenoids, chlorophyll *a*, and chlorophyll *b*. High soil salinity lowers wheat leaf water potential, turgor pressure, and stomata closure, as well as CO_2_ conductance across the stomata, cell wall integrity, oxidative stress, and toxic metabolite synthesis, all of which lead to ultimate plant death [2]. The stomatal pattern of gas conductance is reduced by salt stress, resulting in inadequate CO_2_ supply to wheat plants, which contributes to increased production of reactive oxygen species (ROS) [6].

Osmotic adjustment is a plant’s ability to maintain turgor pressure and water content in the cells despite changes in water potential. This is achieved through the accumulation of solutes, such as sugars and amino acids, in the cells, which increases the osmotic potential and helps to maintain water uptake and turgor pressure. This can help plants to survive in saline soils where the water potential is low, and it allows the plant to maintain a better growth and development [7]. In other words, osmotic and ion toxicity effects were thought to be spatially and temporally separated. This spatial and temporal separation suggested that early salinity stress responses are due to general osmotic or water deficit stress and that sodium-specific responses (i.e., ion sequestration or exclusion) are induced later [8]. Salinity adaptation mechanisms such as osmotic adjustment and cellular exclusion and compartmentalization of Na^+^ ions play a role in alleviating the deleterious effects of salinity [9].

To protect and preserve osmotic stability, cells accumulate proline, which is probably the most widely distributed osmolyte found in plants and other organisms [10]. Proline is a compound that tends to accumulate in response to metabolic salt stress [11]; it is thus important in the osmotic adjustment in plants under stress and serves as an osmoprotectant [12]. A high level of proline in the cytosol reduces the cellular water potential below the external water potential, enhancing the water flow into the cells to maintain cellular water status and plant cell turgidity [13]. Apart from acting as an osmolyte for osmotic adjustment, proline contributes to stabilizing subcellular structures (e.g., proteins and membranes) [14], buffering cellular redox potential against stresses [15,16].

Photosynthesis is the major source of energy that has significant implications for all aspects of plant metabolism and physiology [17]. The redox status of plant cells is mainly determined by photosynthesis, which is why they are at the center of regulatory networks [18]. As a result, the analysis of plant photosynthetic efficiency based on measurements of chlorophyll fluorescence parameters is recommended as an accurate tool for evaluating plant responses to unfavourable photosynthesis environmental conditions and their impact on the plants [19]. Accordingly, the evaluation of the central role of photosynthesis in plant phenotyping is very important [20,21].

Chlorophyll-*a* fluorescence has been described as a re-emission of absorbed light that plants cannot use in the photochemical process of photosynthesis. The inverse relationship between fluorescence kinetics and photosynthesis helps us to understand the biophysical processes of photosynthesis. Measurement of chlorophyll-*a* fluorescence is a valuable non-invasive tool that has been used in eco-physiological studies and extensively used to evaluate the response of plants to environmental stress [22].

The JIP-test is a quick and efficient way to assess the effectiveness of acclimation procedures. It has been used to study the relationship between light-dependent processes and chlorophyll-*a* fluorescence and also when selecting crops for salt and drought tolerance [23–25]. The concept of "energy flux" through thylakoid membranes serves as the basis [26]. The equilibrium between the total energy inflows and outflows for each of the light-collecting complexes studied can be represented by the operationalized simple algebraic equations of this theory, which also reveal the probable distribution of absorbed energy. These equations can be used to explain how the photosystem II (PSII) complexes interact energetically (also known as "grouping", "connectivity", and "overall grouping probability") [14].

The primary goal of this study was to assess the effects of salt stress on 20 wheat genotypes using chemical and physiological properties, including photosynthetic efficiency of plants using chlorophyll-*a* fluorescence measurements. This may help to understand the mechanisms behind the variations in their tolerance and to test which parameters can be used as biomarkers for rapid monitoring of plants growing under salt stress.

## 2. Materials and methods

### 2.1. Plant material and salt treatments

In this study, 20 genotypes were selected from a population of 240 native (Iranian) wheat landraces that had been previously tested for stress resistance by the Agricultural Research Institute. These 20 genotypes were chosen as the best genotypes from the previous study (Table 1). The surface of the seeds was sterilised in 3% H_2_O_2_ for 20 minutes and then ten times in distilled water. In 2018, the experiment was conducted at the Zabol University Research Greenhouse using a randomized complete block design (RCBD) with two replicates. The treatments included two salinity levels of 90 and 120 mM NaCl, with three gradual steps-initiation, and a control treatment. Seedlings were hydroponically cultivated in custom- made plastic trays (Width 60 cm, length 60 cm, and height 20 cm; Luoxi Plastic Products Co., Shandong, China). Ten seeds were placed in each hole before the trays were placed in other rectangular plastic trays with 4.0 L of Hoagland’s solution [27]. These solutions were continuously aerated by electric pumps and renewed every 7 days. Salt treatments were initiated after germination and lasted about 3 weeks. HCl or KOH were used to maintain the pH of the solution at 6.5 throughout the experiment. The study was performed in growth chambers under artificial light (fluorescent lamp photosynthetic photon flux density (PPFD) of 150 μmol m^−2^ s^−1^). NaCl solutions with concentrations of 90 mM and 120 mM were used to feed the corresponding treatments to test the effects of different salinity levels on 20 wheat genotypes. The pH of Hoagland’s nutrient solutions and EC were adjusted to 6–6.5 and 20 mM with HCl or KOH in the control treatment, respectively. Five samples were used for each replication. Samples were stored at -80°C until chemical parameters were measured.

**Table 1:** Information on sensitive and tolerant genotypes.

	Ent	Cid	GID	Taxon	Origcty	Collsite
**Sensitive genotypes**	77	176907	189040	*Triticum aestivum* subsp. aestivum	IRAN	
	81	176948	189193	*Triticum aestivum* subsp. aestivum	IRAN	-
	101	350316	189956	*Triticum aestivum* subsp. aestivum	IRAN	Hamedan
	120	268851	283138	*Triticum aestivum* subsp. aestivum	IRAN	Zanjan
	124	348935	283449	*Triticum aestivum* subsp. aestivum	IRAN	Mashhad
	126	348960	283553	*Triticum aestivum* subsp. aestivum	IRAN	Mashhad
	127	349005	283602	*Triticum aestivum* subsp. aestivum	IRAN	Mashhad
	204	267912	375626	*Triticum aestivum* subsp. aestivum	IRAN	Kerman
	210	267763	375620	*Triticum aestivum* subsp. aestivum	IRAN	Kerman
	213	268362	375743	*Triticum aestivum* subsp. aestivum	IRAN	Kerman
**Tolerant genotypes**	2	299978	319956	-	-	-
	11	178271	187505	Undetermined sp.	IRAN	Saghez
	86	176978	189280	*Triticum aestivum* subsp. aestivum	IRAN	-
	109	177264	190095	*Triticum aestivum* subsp. aestivum	IRAN	Kermanshah
	151	350238	374133	*Undetermined sp*.	IRAN	Ilam
	191	349936	375454	*Triticum aestivum* subsp. aestivum	IRAN	Mashhad
	199	350063	375564	*Triticum aestivum* subsp. aestivum	IRAN	Mashhad
	205	268045	375659	*Triticum aestivum* subsp. aestivum	IRAN	Kerman
	232	179295	375963	*Triticum aestivum* subsp. aestivum	IRAN	Esfahan
	239	350576	2437249	*Triticum aestivum* subsp. aestivum	IRAN	Tehran

### 2.2. Determination of chlorophyll a fluorescence

Chlorophyll fluorescence signals were measured using HandyPEA portable fluorometer (Hansatech Instruments Ltd., Norfolk PE32IJL, England) (Table 2). First, plants were adapted in the dark for at least 30 min using leaf clips (4 mm diameter cross section) and a red LED saturation pulse of 3500 μmol photon m^−2^ s^−1^. Measurements were made in the middle of fully developed leaves of both the control and salt-stressed plants. For each treatment, two measurements were taken on five different plants (10 repetitions as a total for each treatment).

**Table 2:** Definition of selected JIP test parameters [28,29].

JIP parameter	Interpretation
**F**_**0**_	Minimal fluorescence
**F**_**M**_	Maximal fluorescence
**φ**_**P0**_	Maximal quantum yield of the primary photochemical reaction in PSII in dark-adapted samples
**Φ**_**E0**_	Quantum yield of the process of electron transfer from Q_A_-to electron carriers beyond Q_A_-
**ψ**_**E0**_	The probability that the energy of an exciton trapped by active PSII reaction centre (RC) will be utilized for electron transport beyond Q_A_
**Sm**	Тotal electron carriers, per RC, reduced during the time of the induction rise (from F_0_ to F_M_)
**N**	Тurnover number, expressing how many times Q_A_ is reduced until F_M_ is reached
**M**_**0**_	Approximated initial slope (in ms^−1^) of the fluorescence transient normalized to the maximal variable fluorescence. Reflects the maximal rate of initial Q_A_ reduction
**RE**_**0**_ **/RC**	Flux of electrons reaching the end carriers at the acceptor side of PSI as per RC
**ABS/RC**	Absorption flux (exciting PSII antenna Chl *a* molecules) per RC
**RC/CS**_**0**_	Number of active (Q_A_ reducing) PSII reaction centres per cross section
**δRo**	Probability with which an electron from the intersystem electron carriers is transferred to reduce end electron acceptors at the PSI acceptor side
**PI**_**abs**_	Performance index for energy conservation from photons absorbed by PSII until the reduction of intersystem electron acceptors
**PI**_**total**_	Performance index for energy conservation from photons absorbed by PSII until the reduction of PSI end electron acceptors

### 2.3. Determination of photosynthetic pigments

After 21 days, pigment contents were analysed and quantified using techniques proposed by *Harborne* [30]. Fresh leaf samples were washed with deionized water to remove impurities from the surface. Then, 1 g. of the leaf tissue was used to extract pigments with 80% acetone. According to *Knight and Mitchell* [31], the absorbance values for Chl *a*, Chl *b*, and Car. were assessed using a UV-visible spectrophotometer (Model BTS-45, United Kingdom) at three wavelengths, 665, 649, and 470 nm, respectively. And the following formulas were used to determine the outcomes: [Chl *a* (mg g^-1^ FW) = (13.95OD_665_−6.88OD_649_)V/200 W]; [Chl *b* (mg g^-1^ FW) = (24.96OD_649_− 7.32OD_665_)V/200W]; [Car. (mg g^-1^ FW) = (1000OD_470_−2.05Chl a−114.80Chl b) V/ (245×200 W)]

Where; Chl *a*–chlorophyll *a*, Chl *b*–chlorophyll *b*, Car.–carotenoid, V–volume, and W–sample weight, FW–Fresh weight.

### 2.4 Determination of Na^+^ and K^+^

The content of sodium and potassium components in the leaves of wheat genotypes was determined after 21 days of salt stress under hydroponic conditions. They were placed in the oven for drying. The dry weight of the leaves was determined. The dried leaves were placed in Falcon tubes containing 25 mL of a 1% HNO_3_ solution. A solution with a volume of 10 mL was prepared to test K^+^ and Na^+^. Using a flame photometer (JENWAY, model: PFP7, U.K.), leaf samples were subjected to *Shavrukov*, *et al*. [32].

### 2.5. Measurement of Thiobarbituric Acid Reactive Material (TBARM)

The amount of malondialdehyde, the end product and generally stable byproduct of the oxidation process of large molecules, is used to calculate the TBARM value, which is a measure of oxidative stress. In this case, a modified version of the approach of *Harborne* [30] was used. 1 mL of chloroacetic acid (15% w/v) was applied to 0.5 g of homogenised leaves. After the addition of 10 mL of acetone, the mixture was mixed vigorously before centrifugation at 4750 rpm for 15 min. The small precipitate produced by centrifugation was washed with five millilitres of acetone. After vortexing, centrifugation was again performed at the same speed for 10 minutes, repeating the last step four times. The solution was then heated to 100°C for 30 minutes with the addition of 3 millilitres of phosphoric acid (1 wt%) and 1 millilitre of thiobaric acid (0.6 wt%). The reaction was stopped by rapidly cooling the tubes in ice, and the resulting solution’s adsorption amount was measured at 532 and 590 nm using an optical adsorption apparatus (BR Technologies model BT 600). Last but not least, the amount of TBARM in 1 g. fresh weight was measured as Fresh Weight (FW).

### 2.6. Measurement of Lipoxygenase (LOX)

To measure the amount of LOX, 2.5 g of the leaf sample was placed in cold water and then centrifuged (12000 g for 10 minutes). After centrifugation, the top solution was removed, and the rest was purified using the PD-10 gel column. An equal amount of potassium phosphate buffer (pH = 6.6 mm) was added, and sodium and linoleic acid buffer (80 nmol) were added to the homogeneous solution and measured at 234 nm using a spectrophotometer (model BTS-45, United Kingdom) [33].

### 2.7. Measurement of proteins amount

Protein content was measured by *Bradford* [34] method using a spectrophotometer (model BTS-45, United Kingdom) at 595 nm. This method is based on the binding of Comaxi Briant Blue G250 in an acidic reagent to a protein molecule.

### 2.8. Measurement of proline levels

Measurement of proline was performed by the method of *Bates*, *et al*. [35]. 0.1 g. of freshly beaten leaf tissue was poured into 15 mL of Falcons with liquid nitrogen and 10 mL of sulfosalicylic acid 3% was added to the samples. The solvent was centrifuged at 13,000 rpm for 10 minutes. After centrifugation, 2 milliliters of ninhydrin solution were added to the samples. The solution was placed in a steam bath at 100°C for 1 hour. Then, the tubes were placed in a mixture of water and ice for 10 minutes to prevent a further reaction. After heating with the medium, 4 mL of toluene was added to the samples. After vortexing, the absorbance was read at 520 nm using a spectrophotometer (model BTS-45, United Kingdom).

### 2.9. Statistical analysis

The statistix 8.1 programme was used to examine all data using the variance test (One way-ANOVA) [36,37]. With a p-value of *p≤0*.*05*, Fisher’s least significant difference test was used to compare the means of each characteristic within each genotype.

## 3. Results and discussion

### 3.1. Chlorophyll a fluorescence

**Fig 1:**
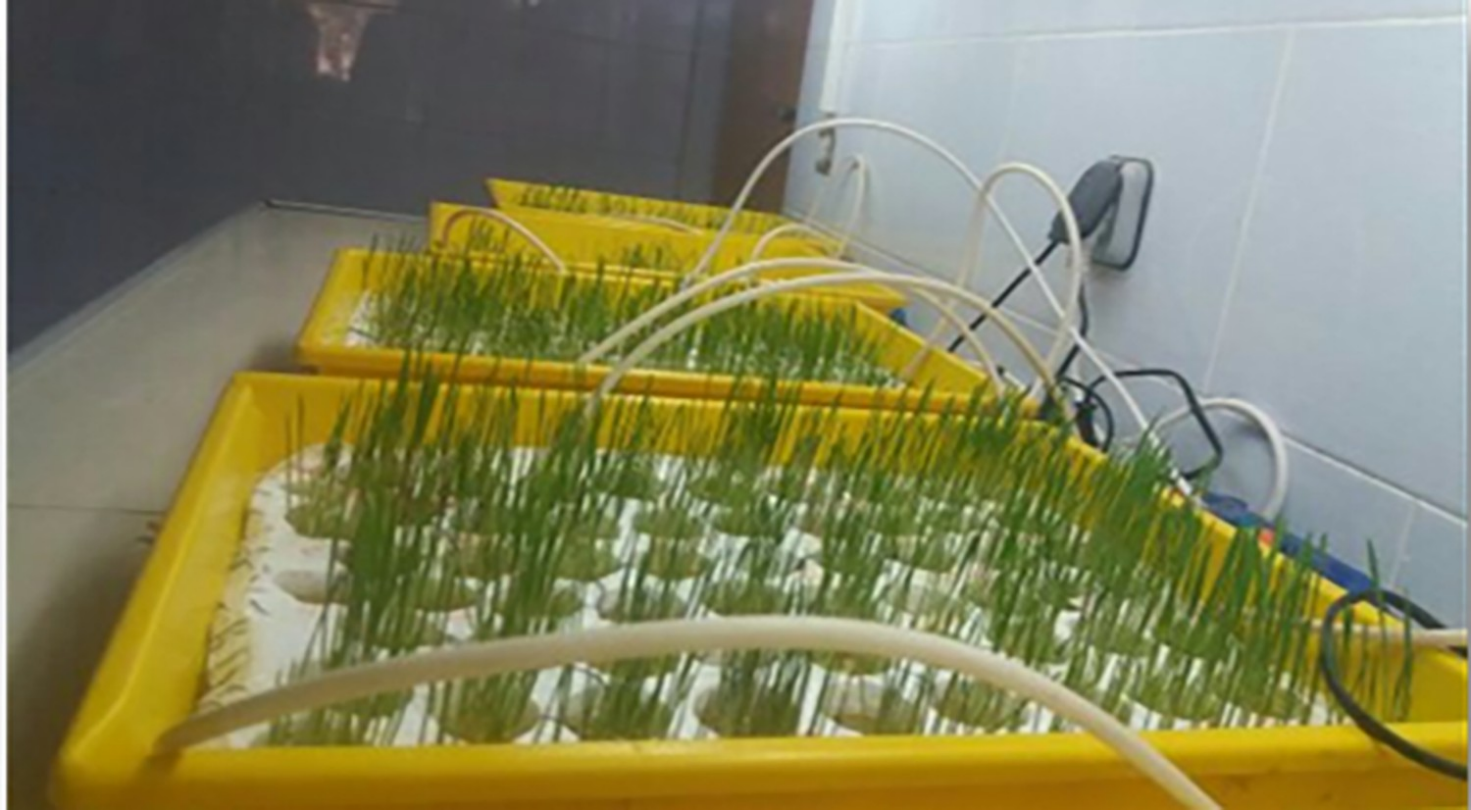
Twenty wheat genotypes (ten sensitive and ten tolerant genotypes) were grown in hydroponic boxes exposed to various concentrations of NaCl.

**Fig 2:**
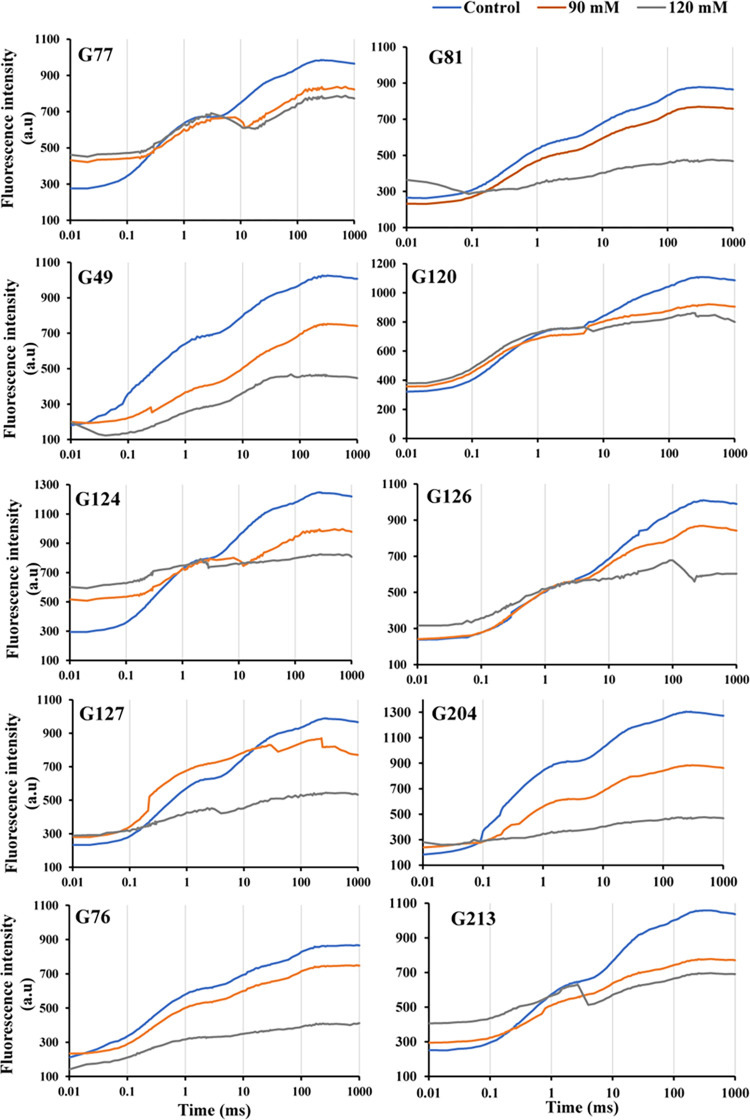
Chlorophyll *a* fluorescence induction curve of 10 sensitive wheat genotypes exposed to various NaCl concentrations after 21 days.

**Fig 3:**
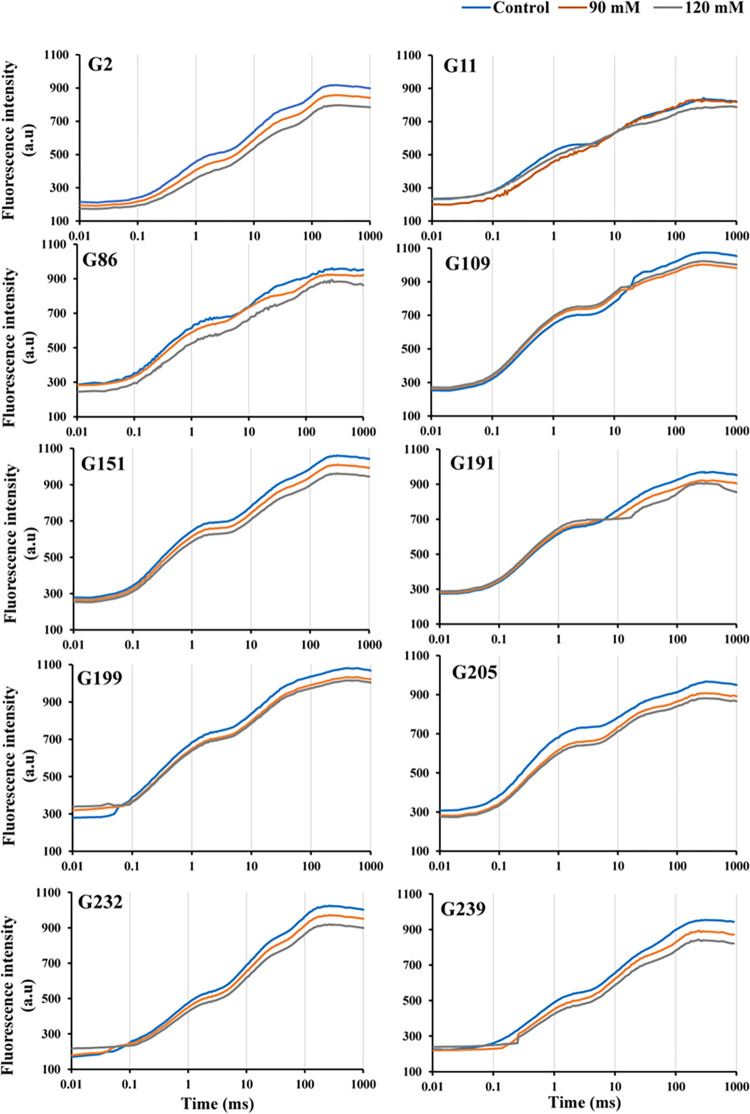
Chlorophyll *a* fluorescence induction curve of 10 tolerant wheat genotypes exposed to various NaCl concentrations after 21 days.

Twenty wheat genotypes (ten sensitive genotypes and ten tolerant genotypes) were selected for salinity tolerance test from the 240 wheat genotypes (Fig 1) using 2 parameters: Total Performance Index (PI_total_) and Performance Index of Absorbance (PI_abs_). Chlorophyll fluorescence induction curves (logarithmic time scale of 0.01 to 1.000 ms) were generated to evaluate the differences between stress and control plants after twenty-one days of stress application (Figs 2 and 3).

**Fig 4:**
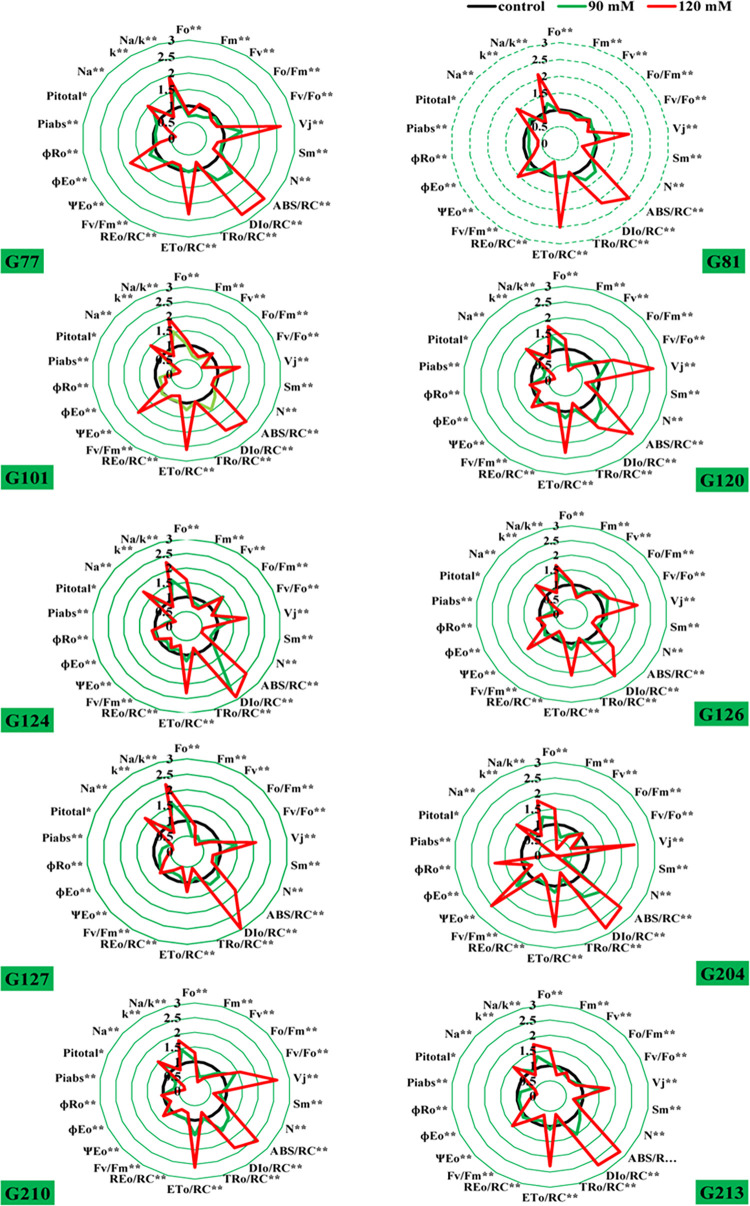
Spider plot presenting the JIP-test parameters (Table 2) calculated from 10 sensitive genotypes of wheat exposed to various concentrations of NaCl after 21 days.

**Fig 5:**
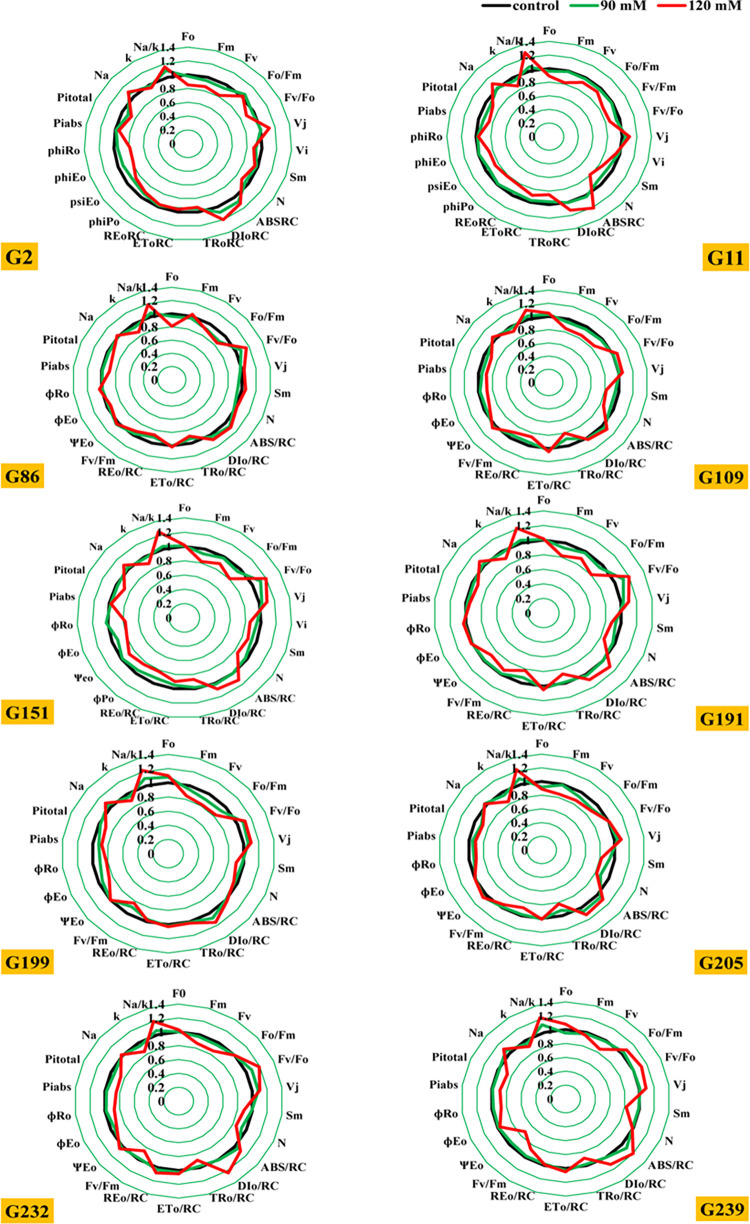
Spider plot presenting the JIP-test parameters (Table 2) calculated from 10 tolerant genotypes wheat exposed to various concentrations of NaCl after 21 days.

To present the obvious figures, the graph was divided into two parts (tolerant and sensitive genotypes), each containing ten graphs of wheat genotypes (Figs 2–5). The effect of different salinity on the transient fluorescence curve of the sensitive genotypes of wheat landraces was very significant after 21 days compared to the tolerant genotypes (Figs 2 and 3). The flat fluorescence curve of the sensitive genotypes revealed that the response of photosynthesis process to salinity stress was quite rapid, and strong (Fig 2). Salinity had a significant negative effect on the 0 and P stages of the curve in the sensitive genotypes (Fig 2). In contrast, salinity showed no significant effect on these bands in the tolerant genotypes (Fig 3).

Applied salt stress resulted in a significant increase in O-J phase in some sensitive genotypes (G77, G124, G126, G127, G210, G210, and G213) as compared to tolerant genotypes (Figs 2 and 3). Under conditions of 120 mM, the J-P phase of the chlorophyll fluorescence transient curves decreased significantly in sensitive genotypes. Additionally, the typical appearance of J-I phase of the OJIP curve was completely lost in all sensitive genotypes. The shape of the chlorophyll fluorescence transient curve was typical in the tolerant genotypes under the same conditions (Fig 3). Moreover, under 90 mM NaCl, the typical J-I phase of the OJIP curve was completely lost in several sensitive genotypes (G120, G124, G204, and G213). Under salt stress, the J-P phase of the chlorophyll fluorescence transient curve was significantly decreased in the sensitive genotypes.

The experiment was conducted not only to evaluate the response of different genotypes to salt stress but also to explore the significance of a rapid fluorescence approach for phenotyping of wheat. Previous studies have shown that physiological parameters such as PI_abs_ and PI_total_, which are directly related to the function of the photosynthetic apparatus, can be used as reliable indicators or biomarkers of salt tolerance in wheat [38].

The spider plot revealed that under salt stress, there was a decrease in F_M_, F_V_, F_V_/F_0_, ET_0_/RCsm, PI_total_, and PI_abs_ as indicated by the parameter’s values (Figs 4 and 5). Compared with the control, an increase in F_0_, V_J_, F_V_/F_0_, DI_0_/ RC, and ABS /RC values was observed. No significant differences were observed between the control and salt-tolerant genotypes in fluorescence indices (fluorescence at time point -F_0_, maximum fluorescence intensity -F_M_), and ratio between photochemical and non-photochemical quantum efficiency -F_V_/F_0_), electron transport quantum yield (φ_E0_), vitality index (power index on an absorbance basis), and specific energy fluxes (ET_0_/ RC and DI_0_/ RC) (Fig 4). Significant differences were detected in sensitive genotypes under salt stress, but a significant decrease in F_V_/F_0_, ET_0_/ RC, F_M_, F_V_, F_V_/F_0_, ET_0_/ RC, Sm, and absorption-based performance (PI_abs_)index and PI_total_, and an increase in V_J_, ABS /RC, and DI_0_/ RC compared with control (Fig 5). In sensitive genotypes, the absorption flux per reaction center (ABS /RC), dissipation energy flux per RC (DI_0_/ RC), minimum fluorescence (F_0_), number of Q_A_ redox conversions to F_M_ (N), size of the pool of electron acceptors on the reducing side of PSII (Sm), and loss of energy absorbed in antennas (ɸDo) were found to vary significantly between 90 mM and 120 mM (Fig 5).

The electron transport chain and chloroplast-based carotenoid and chlorophyll production are two important environmental targets [39]. While phosphorylation and NADP photoreduction occur via the electron transport chain, its electron carriers, and enzymes, chlorophyll and carotenoid production can be linked to the Light Harvest Complexes (LHC) and photosynthetic reaction centres antennae [40]. The JIP assay and its parameters can be used to identify and evaluate the change at these two targets. The light is re-emitted with the help of chlorophyll molecules when returning from excited to unexcited state, which is called chlorophyll fluorescence. It is used as an index of photosynthetic energy conversion in higher plants, bacteria and algae [41]. Excited chlorophyll, which drives photosynthesis, emits the assimilated light energy as heat in non-photochemical suppression or by mission as fluorescence radiation [42]. Because these services are complementary, the study of chlorophyll fluorescence is an important tool in plant research with a wide range of applications [43]. When the total amount of Q_A_ is oxidised, the initial chlorophyll fluorescence at the O band shows the lowest fluorescence yield [44]. The P band corresponds to the state of fully assembled Q_A_ molecules in the moderated state. The J and I bands are measured at 2 and 30 milliseconds, respectively. The transition from stage O to J is brought about by the conversion of Q_A_ to Q_A_^-^ and is associated with the main photochemical processes of PSII. Both the presence of fast and slow reducing PQ centres and the different redox states of PSII reaction centres are reflected in the intermediate step I and final step P. The OJIP transient represents the gradual reduction of the electron transport pool of PSII [45].

The intensity of fluorescence in the OJIP transient curve decreases with increasing NaCl loading concentration, as shown in Figs 2 and 3. The typical polyphasic transient was sensitive to 120 mM salinity treatment in all genotypes. The J-I and I-P phases were decreased under these conditions (Fig 2). While in the tolerant genotypes under the same conditions, the shape of the chlorophyll-*a* fluorescence curve was typical (Fig 3). Also, the O-J phase of the chlorophyll-*a* fluorescence transient curve in sensitive genotypes under salt stress decreases sharply under 90 mM conditions, but it drastically decreased in some sensitive genotypes, including G120, G124, G204, and G213 (Fig 2). The J-I phase of the curve corresponds to the decrease in the secondary electron acceptors Q_B_, plastoquinone (PQ), cytochrome b_6_*f* (Cyt b_6_*f*), and plastocyanin (PC). Also, the typical multiphase transient was saline when treated with 120 mM, and the O-J phase was significantly decreaed under these conditions in all sensitive genotypes except G77, G120, and G126. Different sections of the transient fluorescence curve or OJIP curve show different photosystem events. The I-P phase reflects the rate of ferredoxin reduction and is used as a measure of the relative abundance of PSI compared to PSII, while the J-I phase is responsible for the chlorophyll fluorescence quenching that characterises the activity of the water diffusion complex on the PSII donor side [45,46]. The O-J phase is characterised by the gradual decrease of Q_A_, the main electron acceptor in PSII. When this is not the case, it is related to the proportional size of the final PSI electron acceptor pools [47,48]. The reduction of electron transport on the donor side of PSII to the reaction centres for electron transfer from the water decomposition system to PSI or the shrinkage of the pool size of electron acceptors in PSII (Q_A_, Q_B_ and PQ pools) could be the cause of a decrease in fluorescence yield in phases J, I, and P [41,49].

From the data calculated by the JIP test, shown in Figs 4 and 5, it is evident that for all tolerant genotypes, F_V_/F_M_, the maximum quantum yield of PSII photochemistry, F_V_/F_0_, and the activity of the water-splitting complex on the donor side of PSII were not significantly changed by salinity after 21 days (Fig 5). These studies indicate that salt stress has no effect on electron transfer rates on the donor side of PSII. However, F_V_/F_M_ and F_V_/F_0_ changed significantly in all sensitive genotypes after salinity (Fig 4). It can be said that salt stress significantly negatively affects electron transfer rates on the donor side of PSII [22]. Increasing salinity in the sensitive genotypes showed a significant increase in F_0_. However, photosynthetic apparatus was not significantly changed in the tolerant genotypes, whereas F_M_ significantly decreased in the sensitive genotypes.

There may be a number of reasons for the increased F_0_ levels observed in susceptible genotypes under salt stress. One of them could be an increase in the number of inactive reaction centres in which electrons cannot be transported from the decreasing Q_A_, resulting in higher measured F_0_. The separation of LHC II from the PSII core may have been the cause of poor energy transfer from LHC II to the PSII reaction centre, leading to stronger fluorescence from LHCII [50].

The F_M_ increase reflects the decreased number of nonreducing Q_A_ reaction centers. The F_0_ increase and decrease in maximum fluorescence intensity indicate blockage of electron transport to Q_A_ and result in significantly lower radiation dissipation of the excited states of the photosystem II antennae chlorophylls [51]. The decrease in F_V_/F_M_ may be related to a decrease in photosystem II activity and/or a decrease in photochemical activity, indicating a problem with the functioning of the photosynthetic apparatus [52]. Our results showed that salinity strongly increases the maximum electron transport flux per (ET_0_/ RC), the absorption flux per reaction center (ABS/RC), the dissipation energy flux per (DI_0_/ RC), and the relative variable fluorescence at the J-step (V_J_). Increased salinity magnifies significant differences in chlorophyll-*a* fluorescence parameters such as the quantum yield for reduction of the end electron acceptor on the acceptor side of photosystem I (φ_R0_), the quantum yield of electron transport beyond Q_A_ (φ_E0_), and the probability (at time 0) that a trapped exciton will shift an electron into the electron transport chain beyond Q_A_ (ψ_E0_) in sensitive genotypes.

The total amount of photons captured by chlorophyll molecules in all RCs is multiplied by the total number of active RCs to obtain the effective antenna size of active RCs. The value of ABS /RC increased with increasing salt concentration, which decreased the antenna size of active RCs (Figs 4 and 5).

The kinetics of relative (V_J_) were determined using the formula [V_J_ = F_J_-(F_0_/F_M_)-F_0_] to localise the effect of salt stress in the electron transport chain on the acceptor side of the photosystem II. Relative (V_J_) for photosystem II -units shut down = the fraction of RCs closed in the J stage, expressed as a percentage of the total number of RCs that can be closed [53], F_J_ is the fluorescence at the J stage. Figs 4 and 5 show the results consistent with *Kalaji*, *et al*. [54], *Kalaji*, *et al*. [22], and *Kalaji*, *et al*. [55].

After salinization, the ѱ_E0_ values increased significantly below the values of the stressed samples. Thus, it can be said that the salt stress on the ѱ_E0_ values of the salt-stressed samples was high and the electron supply from the PSII donor side (OEC) was low because the carriers efficiently transferred the electrons to the next step of the electron transport chain. This indicates that salinity had a greater effect on the reduction side of PSII than on the ѱ_E0_-acceptor side, where ѱ_E0_: denotes the reduction of the acceptor side of PSII [41]. Indeed, the parameter ѱ_E0_ is associated with the balance between the efficiency and inefficiency of dark responses after Q_A_ treatment, which was significantly increased by 120 mM treatment. This result suggests that salinity induces the redox reaction after Q_A_ due to the decreased connectivity of electron flow from Q_A_ to Q_B_ [56]. In certain genotypes, the φ_R0_ parameter has a similar trend to the ѱ_E0_ value that characterises the PSII acceptor site. It appears that PSI accepts almost all electrons when the electron supply on the donor side is limited [56]. At increased salinity, the φ_E0_ value decreases very sharply and also shows the highest quantum yield for electron transfer via Q_A_ [57]. Thus, higher φ_E0_ is important for the effectiveness of photosynthetic electron movement in susceptible genotypes of wheat under salt stress (Fig 5). It appears that a greater amount of energy was used to restore Q_A_ in the plants.

After 21 days of salt stress, the parameters sm, N, performance index on an absorption basis (PI_abs_), and performance index (PI_total_) affected salinity significantly more in the sensitive genotypes than in the tolerant ones. Under salt stress, there were very significant decreases in both PI_abs_ and PI_total_ parameters. PI_abs_ is also a parameter that reflects the performance and condition of the photosynthetic apparatus, except that it contains three parameters (RC/ABS, TR_0_/DI_0_, and ET_0_/(TR_0_-ET_0_) [58]. Since these three elements are interrelated, the performance index can more accurately reflect the state of the photosynthetic apparatus [59]. PI_abs_ is extremely sensitive than φ_P0_ and can reflect the effects of stress on the photosynthetic apparatus much better [60]. The PI_abs_ and PI_total_ performance indices were used in this work to evaluate performance to loss of PSI end electron acceptors and to quantify PSII performance. In addition, we found that PI_abs_ and PI_total_ are more sensitive than F_V_/F_M_, because although the trend of PI_abs_ is similar to that of F_V_/F_M_, the degree of change was much greater for PI_abs_ than for F_V_/F_M_ (Figs 4 and 5). Therefore, according to this study, PI_abs_ and PI_total_ may be better parameters to study the change of PSII and measure the power to reduce PSI end electrons during stress than (F_V_/F_M_).

The result of our study based on two parameters, performance index for absorption (PI_abs_) and PI_total_, indicated that the tolerant genotypes of wheat were much more resistant to salt stress than the sensitive genotypes. Accordingly, these photosynthetic traits could be good indicators of wheat adaptation to salinity. Consequently, the measurements for these traits are nonintrusive, rapid, and credible; the approach is quite remarkable.

The activities of the tolerant genotypes showed increased Na^+^ and Na/K levels under salt conditions, but the increase was higher in the sensitive genotypes. Salt concentrations of 90 mM and 120 mM caused a significant decrease in K^+^ and a very significant increase in Na^+^ and Na/K in the sensitive genotypes compared with the control (Fig 5).

### 3.2. Chlorophyll and carotenoids contents

Salinity, genotypes, and their interaction were tested using ANOVA. Fisher’s least significant difference test (*P≤0*.*05*) was used to evaluate the comparison of the relevant interactions. The statistically significant differences by different letters for sensitive genotypes and tolerant genotypes shown in Tables 3 and 4, respectively. Different letters among genotypes in the three salinity levels (One-way ANOVA) indicate significant differences. Salt treatment reduced the chlorophyll content of salt-sensitive genotypes (Table 3) more than that of salt-tolerant genotypes (Table 4).

**Table 3:** Effect of various concentrations of NaCl after 21 days on chlorophyll contents in leaves of 10 sensitive genotypes of wheat.

Genotypes	Salinity levels	Chlorophyll *a* (mg g^-1^ FW)	Chlorophyll*b* (mg g^-1^ FW)	Total Chlorophyll (mg g^-1^ FW)	Carotenoids (mg g^-1^ FW)
**77**	Control	21.81±0.52a	9.30±0.66a	27.17±0.65a	98.47±6.96b
	90 mM	18.77±0.53b	6.68±0.14b	23.50±0.66b	103.05±2.19a
	120 mM	12.98±0.40c	4.97±0.49c	16.24±0.51c	57.84±5.73c
**81**	Control	17.01±1.20a	5.57±0.13a	21.43±1.52a	63.51±4.49a
	90 mM	15.58±0.33b	5.34±0.15a	19.60±0.42b	61.74±5.24b
	120 mM	11.78±1.17c	5.31±0.17a	14.62±1.45c	57.76±2.37c
**101**	Control	19.92±1.41b	6.28±0.44b	25.14±0.71b	86.22±2.07b
	90 mM	24.23±0.51a	8.35±0.18a	30.47±1.29a	91.19±2.58a
	120 mM	11.93±1.18c	6.31±0.62b	14.68±0.21c	72.60±2.26c
**120**	Control	19.15±1.08a	11.15±0.79a	23.42±1.32a	95.676±2.71a
	90 mM	14.27±1.31b	7.50±0.16b	17.56±1.61b	76.28±3.24b
	120 mM	12.61±0.98c	5.85±0.58c	15.63±1.22c	79.23±1.12b
**124**	Control	19.27±0.55a	8.88±0.50a	23.90±1.69a	101.74±7.19a
	90 mM	16.67±0.71b	6.08±0.56b	20.92±0.44b	89.29 ±1.89b
	120 mM	14.43±0.20c	4.80±0.37c	18.18±1.80c	29.37±2.91c
**126**	Control	15.60±0.88b	5.81±0.41a	19.57±1.38b	69.27±1.96b
	90 mM	19.77±1.82a	5.70±0.12a	25.03±0.53a	90.79±3.85a
	120 mM	14.43±1.12b	4.38±0.43a	17.06±1.69c	56.36±0.80c
**127**	Control	18.56±1.31a	5.56±0.39a	23.48±1.66a	66.72±4.72a
	90 mM	12.74±0.27b	4.20±0.36ab	16.06±1.36c	63.65±1.35 ab
	120 mM	17.54±1.74a	3.11±0.13b	22.50±0.92b	61.18±6.06b
**204**	Control	15.38±1.09b	5.60±0.40ab	19.31±0.55b	67.70±4.79b
	90 mM	18.76±0.40a	6.85±0.15a	23.55±1.00a	98.23±8.34a
	120 mM	13.42±1.33c	7.48±0.74a	16.46±0.23c	16.33±0.67c
**210**	Control	19.30±1.36a	5.91±0.17a	24.39±1.72a	79.84±2.26a
	90 mM	16.40±0.35b	6.21±0.26a	20.55±1.74b	80.30±3.41a
	120 mM	13.05±1.29c	4.91±0.07b	16.35±0.67c	19.53±0.28b
**213**	Control	16.49±1.17b	6.97±0.20a	20.55±1.45b	85.91±6.07b
	90 mM	18.56±0.39a	6.93±0.29a	23.26±0.49a	90.7±1.92a
	120 mM	11.09±1.10c	6.03±0.09a	13.62±1.35c	12.98±1.28c

According to Fisher’s least significant difference test at *α = 0*.*05*, different letters indicate a significant difference within each genotype. The average and standard deviation are shown by vertical bars (two repetitions,*P ≤0*.*05*). Where FW–Fresh weight.

**Table 4:** Effect of various concentrations of NaCl after 21 days on chlorophyll contents in leaves of 10 tolerant genotypes of wheat.

Genotypes	Salinity levels	Chlorophyll *a* (mg g^-1^ FW)	Chlorophyll *b* (mg g^-1^ FW)	Total Chlorophyll (mg g^-1^ FW)	Carotenoids (mg g^-1^ FW)
**2**	Control	16±1.13a	5.8±0.04a	21.58±1.53a	67.40±4.77b
	90 mM	17±0.36a	5.6±0.08a	22.36±0.47a	72.43±1.54a
	120 mM	14.8±1.47b	5.3±0.11a	20.86±2.07a	53.30±5.28c
**11**	Control	20.3±1.15a	6.2±0.15a	26.25±1.48a	89.05±5.04ab
	90 mM	21.8±2.00a	5.3±0.15a	27.51±2.53a	93.79±8.62a
	120 mM	16.68±1.30b	5.6±0.17a	22.33±1.74b	86.68±6.74ab
**86**	Control	17±1.20a	7.6±0.05a	24.96±1.76a	78.24±5.53b
	90 mM	18.6±0.39a	6.3±0.09a	24.86±0.53a	82.84±1.76a
	120 mM	15.2±1.50b	6.15±0.13a	21.535±2.13c	66.67±6.60c
**109**	Control	18.37±1.04b	7.2±0.24a	25.657±1.45a	68.58±3.88ab
	90 mM	21.2±1.95a	6.15±0.35a	27.435±2.52a	73.50±6.76a
	120 mM	18.5±1.44b	5.8±0.41a	24.23±1.88b	65.05±5.06b
**151**	Control	17.3±1.22a	7±0.05a	24.53±1.73a	78.58±5.56a
	90 mM	17.9±0.38a	6.65±0.09a	24.55±0.52a	79.75±1.69a
	120 mM	16.89±1.67a	5.83±0.12a	22.72±2.25a	75.01±7.43a
**191**	Control	17.2±0.97a	8.21±0.62a	25.541±1.44a	82.16±4.65b
	90 mM	19±1.75a	7.2±0.61a	26.2±2.41a	96.573±8.88a
	120 mM	16.8±1.31a	6.5±0.60a	23.63±1.77a	77.35±6.02b
**199**	Control	17.78±1.26a	7.19±0.71a	24.97±0.53a	76.54±5.41a
	90 mM	17.9±0.38a	6.86±0.29a	24.76±2.30a	81.47±1.73a
	120 mM	16.98±1.68a	6.21±0.22a	23.19±1.40a	73.14±7.24ab
**205**	Control	17.38±0.98a	7.31±0.05a	24.69±2.23a	71.493±4.04a
	90 mM	18.01±1.66a	6.3±0.09a	24.31±1.77a	73.480±6.75a
	120 mM	16.8±1.31a	5.9±0.13a	22.7±0.66a	71.47±5.56a
**232**	Control	13.8±0.39a	9±0.45a	23.18±1.01a	79.448±2.25ab
	90 mM	14.2±0.60a	8.8±0.50a	23.8±0.28a	86.16±3.66a
	120 mM	12.6±0.18a	6.71±0.43ab	19.63±1.96b	76.1±1.08b
**239**	Control	18.68±1.32a	8.6±0.61a	27.73±0.59a	79.38±5.61ab
	90 mM	19.1±0.41a	8.23±0.70a	27.63±2.53a	82.97±1.76a
	120 mM	17.6±1.74a	7.51±0.31a	25.51±1.84a	65.66±6.50b

According to Fisher’s least significant difference test at *α = 0*.*05*, different letters indicate a significant difference within each genotype. The average and standard deviation are shown by vertical bars (two repetitions,*P ≤0*.*05*). Where FW–Fresh weight.

Since carotenoids and chlorophylls are key components of the photosynthetic machinery, their role in light energy collection, membrane stability, and energy transfer has been intensively studied. In general, the levels of chlorophyll (*a* and *b*), total chlorophyll, and carotenoids were lower in non-stressed plants than in salt-stressed plants compared to the control. However, plants treated with 120 mM salinity were significantly different from control plants. This difference was not significant in the tolerant genotypes. In contrast, the tolerant genotypes had higher chlorophyll and carotenoid contents than the sensitive genotypes at both salinity levels at all stages. This was probably due to inhibition of ribulose-1,5-bisphosphate enzyme and the structural destruction of the chloroplast and photosynthetic apparatus, ultimately resulting in a decrease in photosynthetic pigments such as chlorophyll, carotenoids, and CSI [61,62]. Similar results were also observed in wheat plants under high salt stress, with reductions in photosynthetic pigments, photosynthetic rate, stomatal conductance, and CO_2_ intake [63].

This study showed a very significant reduction in carotenoid content when treated with 90 mM and 120 mM salt stress in the sensitive genotypes. In contrast, these changes were insignificant in the tolerant genotypes. Carotenoids are responsible for quenching singlet oxygen [64]. Therefore, their comparable content in a cultivar may determine its relative tolerance. *Hamada* [65] reported that the decrease in chlorophyll content was due to the increase in chlorophyll enzyme activity and the instability of protein complexity of pigments. Carotenoids are important for plants under stress because they play a significant role as precursors in signaling during plant expansion under environmental stress, so they are very important for photoprotection of photosynthesis [66]. This result is confirmed by *Shah*, *et al*. [67]. They also found that chlorophyll and carotenoids decrease with reduced salinity. The decrease in pigment amounts and ratios in this study is also consistent with the results of *Pastuszak*, *et al*. [68], who observed that salt-tolerant wheat genotypes produced less chlorophyll *a*, chlorophyll *b*, carotenoids, and lower chlorophyll *a* to *b* ratio with increasing NaCl.

### 3.3. Na^+^ and K^+^

One of the biggest global issues negatively impacting agricultural yields is salinity. Water stress, cytotoxicity caused by excessive uptake of ions such as sodium (Na^+^) and chloride (Cl^-^), and imbalanced nutrient ratios hinder plant growth and development. Due to the production of reactive oxygen species (ROS), this is also often accompanied by oxidative stress [69].

In this study, salt caused a decrease in K^+^ content in all wheat genotypes (Figs 4 and 5). The K^+^ deficiency was caused by the presence of too much Na^+^ in the growth media, which is known to have a negative effect on K^+^ absorption in plants [70]. Due to the role of K^+^ in osmotic control and competition with Na^+^ [71], salt tolerance is correlated with K^+^ concentration [72]. In general, plants control K^+^ uptake and stop Na^+^ efflux into the cell by maintaining the optimal K/Na ratio in the cytosol. The transport mechanisms involved in the use of Na^+^ as an osmotic solution have received the most attention in research evaluating plant responses to salt stress [73]. Sodium and potassium compete for absorption through a common transport pathway. This competition is successful because Na^+^ concentration is often much higher than K^+^ concentration in saline conditions. Moreover, it has been argued that most plants are susceptible to salt stress because we are unable to keep Cl and Na^+^ out of transpiration currents [74].

Therefore, plants could show greater tolerance by limiting the uptake of harmful ions or maintaining normal nutrient ion levels, which has been done. Uptake mechanisms that discriminate similar ions such as Na^+^ and K^+^ could be critical selection factors for salt tolerance in wheat genotyping and breeding for best nutrient uptake under salt stress.

### 3.4 Indicator of cellular oxidation degree of thiobarbituric acid reactive material (TBARM)

Lipids are the main source of energy for cells [75]. They are the basic mechanism for cell membrane assembly and act as a sensitive insulator between organs and hormones [76]. Salinity resistance and water stress are related to the two lipid layers containing unsaturated fatty acids, which maintain membrane stability in the presence of sugar stress (trehalose. Membrane lipid peroxidation is a combination of malondialdehyde (MDA), propane, butanal, hexane, heptanal, and propanedimethylacetal. These examples are used to determine the amount of membrane lipid peroxidation; an increase in lipid peroxidation is considered to indicate greater oxidative stress. Lipid peroxidation of membranes is a sign of oxidative stress. At the same time, the TBARM assay, which measures malondialdehyde, can be evaluated as an indicator of oxidation levels at the cellular and molecular levels [77].

The application of 120 mM showed a significant downward trend in susceptible genotypes (Table 5), but in resistant genotypes, this index continued to increase from 90 mM to 120 mM with increasing salt stress (Table 6). Since this oxidation index is measurable at the cellular and molecular levels, it can be said that the decreasing trend of this index indicates cell death.

**Table 5:** Effect of various concentrations of NaCl after 21 days on TBARM, LOX, protein, and proline in leaves of 10 sensitive genotypes of wheat.

Genotypes	Salinity levels	TBARM (mg MAD kg^-1^)	LOX (μmol min^−1^g^−1^)	Protein (mg g^-1^ FW)	Proline (μmol L^-1^)
**77**	Control	2.5±0.07c	3.8±0.07c	1.5±0.04c	1.3±0.04c
	90 mM	7.8±0.06b	4.3±0.14b	4.4±0.07a	4.1±0.14a
	120 mM	9.2±0.07a	5±0.21a	3.5±0.07b	3.7±0.20b
**81**	Control	3.3±0.04c	2.9±0.07c	2.5±0.06c	1.45±0.15c
	90 mM	6.9±0.07b	4.7±0.14a	3.6±0.08b	2.4±022b
	120 mM	10±0.14a	4±0.21b	4.3±0.42a	3.8±0.15a
**101**	Control	3.5±0.07c	3.3±0.14c	3.2±0.14c	0.9±0.08c
	90 mM	7.2±0.14b	4±0.14b	4.4±0.14a	2.2±0.07b
	120 mM	12.5±0.14a	4.5±0.21a	3.9±0.15b	3.1±0.13a
**120**	Control	3±0.07c	4±0.21b	2.8±0.04b	1.2±0.14c
	90 mM	9±0.14b	4.2±0.28ab	3.8±0.21a	3.8±0.15a
	120 mM	12±0.15a	4.4±0.14a	4.2±0.28a	3.2±0.16b
**124**	Control	2.9±0.06c	3.4±0.07b	1.5±0.05c	1.54±0.17c
	90 mM	8.4±0.16b	5±0.28a	3.4±0.06b	3.9±0.18a
	120 mM	11±0.15a	4.5±0.23a	3.6±0.08a	3.5±0.15b
**126**	Control	3.7±0.07c	2.8±0.21c	1.1±0.04c	1.32±0.07c
	90 mM	7.9±0.13b	5.9±0.20a	2.9±0.09a	3±0.12b
	120 mM	11.1±0.12a	5.1±0.28b	2.4±0.04b	3.9±0.05a
**127**	Control	2.56±0.06c	3.8±0.13b	2.4±0.07c	1.7±0.05c
	90 mM	7.4±0.08b	6.2±0.15a	4.3±0.04a	4±0.07a
	120 mM	10.5±0.09a	5.7±0.17a	3.5±0.07b	3.7±0.15b
**204**	Control	3.17±0.03c	3.1±0.13c	3.1±0.07c	1.1±0.09c
	90 mM	8.4±0.07b	7±0.27a	4.5±0.07a	3.1±0.16b
	120 mM	10.8±0.14a	5.4±0.21b	3.7±0.14b	4±0.19a
**210**	Control	3.8±0.07c	3.5±0.22c	3.3±0.14c	1.25±0.07c
	90 mM	8±0.07b	5.1±0.23a	5.1±0.33a	2.44±0.12b
	120 mM	12.1±0.01a	4.8±0.18b	4±0.25b	3.3±0.17a
**213**	Control	3.21±0.07c	4±0.12c	3.23±0.24b	1.38±0.18c
	90 mM	7.9±0.14b	6.1±0.20a	4.82±0.27a	3±0.19b
	120 mM	10±0.16a	5.3±0.21b	3.53±0.21b	3.45±0.16a

According to Fisher’s least significant difference test at *α = 0*.*05*, different letters indicate a significant difference within each genotype. The average and standard deviation are shown by vertical bars (two repetitions, *P ≤0*.*05*). Where MAD–malonaldehyde, FW–Fresh weight.

**Table 6:** Effect of various concentrations of NaCl after 21 days on TBARM, LOX, protein, and proline in leaves of 10 tolerant genotypes of wheat.

Genotypes	Salinity levels	TBARM (mg MAD kg^-1^)	LOX (μmol min^−1^g^−1^)	Protein (mg g^-1^ FW)	Proline (μmol L^-1^)
**2**	Control	2.2±0.14c	4.2±0.28c	1.1±0.04c	2±0.05c
	90 mM	5.4±0.07b	8±0.35b	4.9±0.24b	4.1±0.15b
	120 mM	7.2±0.14a	9.8±0.14a	6.1±0.30a	5.8±0.25a
**11**	Control	3.1±0.14b	3.7±0.14c	2.8±0.21c	2.5±0.14c
	90 mM	4.9±0.14a	6.9±0.07b	5.1±0.27b	4.5±0.16b
	120 mM	5.2±0.28a	10.7±0.99a	6.9±0.28a	5.2±0.23a
**86**	Control	3.2±0.07c	3.8±0.07c	3.2±0.19c	2.5±0.12c
	90 mM	4.8±0.07b	7.8±0.07b	4.8±0.21b	5.5±0.27b
	120 mM	5.5±0.14a	11.4±0.57a	7.1±0.20a	6.4±0.30a
**109**	Control	2.7±0.04c	4.7±0.42c	3.7±0.18c	2.75±0.09c
	90 mM	4.9±0.03b	7.2±0.28b	5.5±0.26b	5.1±0.17b
	120 mM	6.6±0.06a	12.2±0.28a	6.1±0.30a	7.8±0.33a
**151**	Control	2.4±0.08c	5±0.07c	2.9±0.14c	2.2±0.08c
	90 mM	5.3±0.07b	7. 4±0.07b	6±0.25b	7±0.36a
	120 mM	5.8±0.04a	9.9±0.14a	7.65±0.32a	6.5±0.32b
**191**	Control	3.1±0.14c	4.5±0.21c	4±0.20b	3±0.14c
	90 mM	4.7±0.07b	6.8±0.14b	7.1±0.35a	6.5±0.30a
	120 mM	6.4±0.07a	11.7±0.42a	6.8±0.36a	5.2±0.28b
**199**	Control	3±0.07c	3.9±0.21c	1.9±0.13c	1.9±0.15c
	90 mM	5±0.14b	8.5±0.21b	5.4±0.16b	6.5±0.28a
	120 mM	8.4±0.07a	12.1±0.14a	6.4±0.23a	5.8±0.24b
**205**	Control	2.8±0.04c	4.1±0.14c	2.7±0.10b	1.4±0.08c
	90 mM	5.1±0.14b	8.2±0.28b	6.7±0.27a	4.7±0.21b
	120 mM	5.4±0.07a	10.4±0.07a	7±0.34a	5.9±0.22a
**232**	Control	3.1±0.07c	4±0.14c	2.4±0.014c	2.1±0.14c
	90 mM	4.9±0.06b	6.9±0.07b	5.8±0.25b	7.2±0.36a
	120 mM	5.7±0.07a	12.6±0.28a	6.7±0.28a	6.8±0.29b
**239**	Control	3.5±0.10c	4.5±0.14c	3.5±0.18c	1.25±0.10c
	90 mM	5.3±0.04b	7.2±0.07b	6.1±0.29b	5.4±0.23b
	120 mM	6.9±0.04a	10.8±0.07a	7.2±0.33a	7.1±035a

According to Fisher’s least significant difference test at *α = 0*.*05*, different letters indicate a significant difference within each genotype. The average and standard deviation are shown by vertical bars (two repetitions, *P ≤0*.*05*). Where MAD–malonaldehyde, FW–Fresh weight.

### 3.5. Lipoxygenase (LOX)

One of the most important enzyme systems at the interface by changing the lipid of cell membranes is the LOX enzyme system [78]. This enzyme controls the reaction of the connection between oxygen molecules and unsaturated fatty acids and the formation of unsaturated fatty acid hydroxides [79]. The oxidation of fatty acids by the activity of this enzyme leads to the formation of oxygen free radicals [80].

According to the results of this study, the effect of salt stress on the accumulation of LOX was significant (Tables 5 and 6), indicating that salt stress has a great effect on the oxidative property of LOX. A high LOX index indicates the abundance of reactive oxygen radicals and the intensity of the oxidative process. In general, stress increases the level of oxygen free radicals in plant cells, such as hydrogen peroxide, and the resulting hydrogen peroxide increases the level of the enzyme LOX in plant cells [81]. This enzyme catalyzes unsaturated and long-chain fatty acids containing a cis bond. Linoleic acid and linolenic acid are the most highly unsaturated fatty acids in plant cell structure, providing an ideal starting material for the activity of this enzyme [82]. Toxic concentrations of reactive oxygen radicals cause severe damage to protein structures, inhibition of the activity of various enzymes in metabolic pathways, and consequent oxidation of macromolecules such as lipids and DNA. The exacerbation and persistence of these adverse events can lead to cell death [83].

### 3.6. Protein

The results of data analysis showed that salinity stress in the Tables 5 and 6 and its interaction with salinity stress were very significant for protein adjectives. Protein synthesis changes in response to environmental stresses such as salt, heat shock, anaerobic conditions, drought, osmotic shock, wounding, and cold stress [84]. Such stresses increased the synthesis of some proteins and decreased the synthesis of others. It appears that the proteins induced by salinity effectively tolerated this stress.

According to the results of average protein comparisons, protein levels increased with increasing salinity stress in all genotypes (Tables 5 and 6). However, with increasing salinity up to 120 mM, this increase was not stable in the susceptible genotypes, so that in these genotypes the level of spreading decreased from 90 mM to 120 mM with increasing salt stress (Table 5). However, an increasing trend was still observed in the resistant genotypes (Table 6). Proteins that accumulate in plants under salinity stress serve as nitrogen reserves in osmotic regulation. In response to salinity stress, proteins can be newly formed or institutionalized in concentration [84]. There are low concentrations, and when plants are exposed to repair and repair damage and salt stress, their concentration increases [85]. In osmotic or ionic stress, elevated stress proteins are essential for cell survival at the level of metabolic inhibition [86]. Other osmotic and physiological adaptations, such as changes in root and shoot development and transpiration, may be involved in these tactics, suggesting that proteins are resynthesized in plants under salt stress [87].

Salt stress leads to quantitative and qualitative changes in the amount of soluble proteins [88]. Proteins that increase in plants under salt stress may be a form of nitrogen storage that is later used by the plant [89]. Apples may play a role in osmotic adaptation, such as the production of pseudo-osmotic proteins or proteins, or they may alter the structure of the cell wall. These proteins may be synthesized in response to salt stress, or perhaps there are structures of concentrations low and high [90].

In this study, the concentration of leaf soluble proteins increased with increasing salt concentration in all genotypes (Tables 5 and 6). It can be said that antiperspirants increase antioxidant activity and also prevent protein degradation, thus increasing protein levels. Many proteins induced by salt stress are molecular chaperones, and some of them are synthesized to counteract the oxidative stress caused by salinity and prevent the destruction of structural and functional proteins by oxidative stress [91].

However, this changed in the sensitive genotypes, so that the control group had the lowest protein level and the salinity group had the highest protein level, 90 mM (Table 5). The significant reduction in protein content in the sensitive genotypes can be attributed to both protein degradation and reduced protein synthesis. Decreased water potential in leaves appears to lead to a sharp decrease in polyribosomes and monoribosomes, implying a decrease in protein synthesis. Oxygen-free radicals, which have a high composition of proteins, also lead to their oxidation [92].

### 3.7. Proline

The mean comparison test (Tables 5 and 6) shows that with increasing salinity, proline accumulation increases in all genotypes.

In plant stress physiology, the accumulation of compatible solutes is generally believed to play a role in maintaining osmotic balance in cells [93,94]. For example, proline accumulation in plants increases salt resistance [95]. As salinity increased from 90 mM to 120 mM, proline significantly decreased in susceptible genotypes (Table 5), whereas it continued to increase in resistant genotypes as salinity increased (Table 6). Therefore, the embryo may play the role of proline in protecting against osmosis, which increases during plant growth. It helps to stabilise the membrane and reduce the effects of NaCl on cell membrane degradation. It is important for regulating osmotic potential, removing free radicals, and preventing denaturation of macromolecules in cellular pH when proline is stressed. Proline also acts as a nitrogen and carbon source for plants under extreme stress and increases the stress tolerance of plants [96]. Therefore, it can be said that proline is the most effective regulator of osmotic pressure of higher plants under stress salinity. Under stress conditions, proline plays a role in maintaining membrane structure, creating osmotic compatibility, and maintaining the structure of enzymes in the cell [97].

Therefore, genotypes that produce more proline may be more resistant to stress [98]. Considering that salt stress is one of the most important stress factors affecting plant performance, understanding the mechanisms that plants use when exposed to stress is of great importance for plant breeding research. Plants need to enhance resistance mechanisms such as reactive oxygen radical removal and cellular defence system to maintain equilibrium under stress [99].

## 4. Conclusions

Our study found that salinity had a significant effect on the photochemical reaction of the photosynthetic apparatus in wheat plants, as indicated by changes in parameters of chlorophyll-a fluorescence. Additionally, the results showed that salt stress had similar mechanisms of action on the light-dependent photosynthetic phase in nearly all genotypes, but certain wheat genotypes were particularly sensitive to salt stress when it comes to PSII. Salinity stress led to an increase in energy dissipation and damage to the oxygen evolving complex and reaction centers in plants. However, to cope with and survive this stress, plants boost the activity of antioxidant enzymes that break down or remove harmful compounds from the cell. As a result, their levels in the plant increase during salt stress.

Therefore, molecular studies need to be conducted and linked to morphological, physiological, and biochemical traits to understand the processes behind the effects of salt stress on plant growth and photosynthetic efficiency. Nevertheless, we recommend the use of prompt chlorophyll fluorescence parameters as bio-indicators for a quick survey of tolerance in wheat plants under salinity.

## References

[1] Arzani A, Ashraf M. Cultivated ancient wheats (Triticum spp.): A potential source of health‐beneficial food products. Comprehensive Reviews in Food Science Food Safety. 2017;16(3):477–88. doi: 10.1111/1541-4337.12262 33371554

[2] Pramila M, Kumar U, Yadav L. Promoting & reinvigorating agri-horti, technological innovations [pragati-2019]. International Journal of Chemical Studies. 2019;(SP6):592–5.

[3] Zhou H, Finkemeier I, Guan W, Tossounian MA, Wei B, Young D, et al. Oxidative stress‐triggered interactions between the succinyl‐and acetyl‐proteomes of rice leaves. Plant, cell environment. 2018;41(5):1139–53. doi: 10.1111/pce.13100 29126343

[4] Arzani A. Improving salinity tolerance in crop plants: a biotechnological view. In Vitro Cellular & Developmental Biology-Plant. 2008;44(5):373–83. 10.1007/s11627-008-9157-7.

[5] Munns R, Tester M. Mechanisms of salinity tolerance. Annual review of plant biology. 2008;59:651. doi: 10.1146/annurev.arplant.59.032607.092911 18444910

[6] Acosta-Motos JR, Ortuño MF, Bernal-Vicente A, Diaz-Vivancos P, Sanchez-Blanco MJ, Hernandez JA. Plant responses to salt stress: adaptive mechanisms. Agronomy. 2017;7(1):18. 10.3390/agronomy7010018.

[7] Flower DJ, Ludlow MM. Variation among accessions of pigeonpea (Cajanus cajan) in osmotic adjustment and dehydration tolerance of leaves. Field Crops Research. 1987;17(3–4):229–43. 10.1016/0378-4290(87)90037-2.

[8] Van Zelm E, Zhang Y, Testerink C. Salt tolerance mechanisms of plants. Annual review of plant biology. 2020;71:403–33. doi: 10.1146/annurev-arplant-050718-100005 32167791

[9] Arzani A, Ashraf M. Smart engineering of genetic resources for enhanced salinity tolerance in crop plants. Critical Reviews in Plant Sciences. 2016;35(3):146–89. 10.1080/07352689.2016.1245056.

[10] Liang XW, Zhang L, Natarajan SK, Becker DF. Proline mechanisms of stress survival. Redox Signal. 2013;19:998–1011. doi: 10.1089/ars.2012.5074 23581681 PMC3763223

[11] Hosseinifard M, Stefaniak S, Ghorbani Javid M, Soltani E, Wojtyla Ł, Garnczarska M. Contribution of Exogenous Proline to Abiotic Stresses Tolerance in Plants: A Review. International Journal of Molecular Sciences. 2022;23(9):5186. doi: 10.3390/ijms23095186 35563577 PMC9101538

[12] Joseph EA, Radhakrishnan VV, Mohanan KV. A study on the accumulation of proline-an osmoprotectant amino acid under salt stress in some native rice cultivars of North Kerala, India. Universal Journal of Agricultural Research. 2015;3(1):15–22.

[13] Hasegawa PM, Bressan RA, Zhu J-K, Bohnert HJ. Plant cellular and molecular responses to high salinity. Annual review of plant biology. 2000;51(1):463–99. doi: 10.1146/annurev.arplant.51.1.463 15012199

[14] Stirbet A. Excitonic connectivity between photosystem II units: what is it, and how to measure it? Photosynthesis research. 2013;116(2):189–214. 10.1007/s11120-013-9863-9. 23794168

[15] Miller G, Honig A, Stein H, Suzuki N, Mittler R, Zilberstein A. Unraveling Δ1-pyrroline-5-carboxylate-proline cycle in plants by uncoupled expression of proline oxidation enzymes. Journal of Biological Chemistry. 2009;284(39):26482–92. 10.1074/jbc.M109.009340. 19635803 PMC2785336

[16] Servet C, Ghelis T, Richard L, Zilberstein A, Savoure A. Proline dehydrogenase: a key enzyme in controlling cellular homeostasis. Frontiers in Bioscience-Landmark. 2012;17(2):607–20. doi: 10.2741/3947 22201764

[17] Parry MA, Hawkesford MJ. An integrated approach to crop genetic improvement F. Journal of integrative plant biology. 2012;54(4):250–9. 10.1111/j.1744-7909.2012.01109.x. 22348899

[18] Scheibe R, Backhausen JE, Emmerlich V, Holtgrefe S. Strategies to maintain redox homeostasis during photosynthesis under changing conditions. Journal of experimental botany. 2005;56(416):1481–9. doi: 10.1093/jxb/eri181 15851411

[19] Kuckenberg J, Tartachnyk I, Noga G. Temporal and spatial changes of chlorophyll fluorescence as a basis for early and precise detection of leaf rust and powdery mildew infections in wheat leaves. Precision agriculture. 2009;10(1):34–44. 10.1007/s11119-008-9082-0.

[20] Berberan-Santos MN, Bodunov EN, Valeur B. Luminescence decays with underlying distributions of rate constants: General properties and selected cases. Fluorescence of Supermolecules, polymers, and Nanosystems: Springer; 2007. p. 67–103.

[21] Santos C, Ribeiro R, Magalhães Filho J, Machado D, Machado E. Low substrate temperature imposes higher limitation to photosynthesis of orange plants as compared to atmospheric chilling. Photosynthetica. 2011;49(4):546–54. 10.1007/s11099-011-0071-6

[22] Kalaji HM, Jajoo A, Oukarroum A, Brestic M, Zivcak M, Samborska IA, et al. Chlorophyll a fluorescence as a tool to monitor physiological status of plants under abiotic stress conditions. Acta physiologiae plantarum. 2016;38(4):102. 10.1007/s11738-016-2113-y.

[23] Clavel D, Diouf O, Khalfaoui JL, Braconnier S. Genotypes variations in fluorescence parameters among closely related groundnut (Arachis hypogaea L.) lines and their potential for drought screening programs. Field crops research. 2006;96(2–3):296–306. 10.1016/j.fcr.2005.07.012.

[24] Oukarroum A, El Madidi S, Schansker G, Strasser RJ. Probing the responses of barley cultivars (Hordeum vulgare L.) by chlorophyll a fluorescence OLKJIP under drought stress and re-watering. Environmental and Experimental Botany. 2007;60(3):438–46. 10.1016/j.envexpbot.2007.01.002.

[25] Petkova V, Denev ID, Cholakov D, Porjazov I. Field screening for heat tolerant common bean cultivars (Phaseolus vulgaris L.) by measuring of chlorophyll fluorescence induction parameters. Scientia Horticulturae. 2007;111(2):101–6. 10.1016/j.scienta.2006.10.005.

[26] Strasser RJ, Srivastava A, Tsimilli-Michael M. The fluorescence transient as a tool to characterize and screen photosynthetic samples. Probing photosynthesis: mechanisms, regulation and adaptation 2000. p. 445–83.

[27] Hoagland DR, Arnon DI. The water-culture method for growing plants without soil. Circular California agricultural experiment station. 1950;347(2nd edit).

[28] Goltsev V, Kalaji H, Paunov M, Bąba W, Horaczek T, Mojski J, et al. Variable chlorophyll fluorescence and its use for assessing physiological condition of plant photosynthetic apparatus. Russian journal of plant physiology. 2016;63(6):869–93. 10.1134/S1021443716050058.

[29] Tsimilli-Michael M. Revisiting JIP-test: An educative review on concepts, assumptions, approximations, definitions and terminology. Photosynthetica. 2020;58:275–92. 10.32615/ps.2019.150.

[30] Harborne J. Methods of plant analysis. Phytochemical methods: Springer; 1984. p. 1–36.

[31] Knight S, Mitchell C. Enhancement of lettuce yield by manipulation of light and nitrogen nutrition. HortScience: a publication of the American Society for Horticultural Science. 1983;108(5):750–4. 10.21273/JASHS.108.5.750. 11542284

[32] Shavrukov Y, Langridge P, Tester M. Salinity tolerance and sodium exclusion in genus Triticum. Breeding Science. 2009;59(5):671–8. 10.1270/jsbbs.59.671.

[33] Xu B, Miao Wj, Guo K, Hu Qs, Li B, Dong Y. An improved method to characterize crude lipoxygenase extract from wheat germ. Quality Assurance Safety of Crops Foods. 2012;4(1):26–32. 10.1111/j.1757-837X.2011.00120.x.

[34] Bradford MM. A rapid and sensitive method for the quantitation of microgram quantities of protein utilizing the principle of protein-dye binding. Analytical biochemistry. 1976;72(1–2):248–54. doi: 10.1006/abio.1976.9999 942051

[35] Bates LS, Waldren RP, Teare I. Rapid determination of free proline for water-stress studies. Plant soil. 1973;39(1):205–7. 10.1007/BF00018060.

[36] Gomez KA, Gomez AA. Statistical procedures for agricultural research: John Wiley & Sons; 1984.

[37] Snedecor GW, Cochran WG. Statistical methods, 8thEdn. Ames: Iowa State University Press Iowa. 1989;54:71–82.

[38] Oukarroum A, Schansker G, Strasser RJ. Drought stress effects on photosystem I content and photosystem II thermotolerance analyzed using Chl a fluorescence kinetics in barley varieties differing in their drought tolerance. Physiologia Plantarum. 2009;137(2):188–99. doi: 10.1111/j.1399-3054.2009.01273.x 19719481

[39] Rochaix J-D. Regulation of photosynthetic electron transport. Biochimica et Biophysica Acta -Bioenergetics. 2011;1807(3):375–83. 10.1007/0-306-48148-0_30. 21118674

[40] Dayan FE, Zaccaro MLdM. Chlorophyll fluorescence as a marker for herbicide mechanisms of action. Pesticide Biochemistry Physiology. 2012;102(3):189–97. 10.1016/j.pestbp.2012.01.005.

[41] Tóth SZ, Schansker G, Garab G, Strasser RJ. Photosynthetic electron transport activity in heat-treated barley leaves: the role of internal alternative electron donors to photosystem II. Biochimica et Biophysica Acta -Bioenergetics. 2007;1767(4):295–305. doi: 10.1016/j.bbabio.2007.02.019 17412308

[42] Ruban AV. Nonphotochemical chlorophyll fluorescence quenching: mechanism and effectiveness in protecting plants from photodamage. Plant physiology. 2016;170(4):1903–16. doi: 10.1104/pp.15.01935 26864015 PMC4825125

[43] Lu Y, Miao X, Song Q, Peng S, Duan B. Morphological and ecophysiological plasticity in dioecious plant Populus tomentosa under drought and alkaline stresses. Photosynthetica. 2018;56(4):1353–64. 10.1007/s11099-018-0846-0.

[44] Kramer DM, Johnson G, Kiirats O, Edwards GE. New fluorescence parameters for the determination of QA redox state and excitation energy fluxes. Photosynthesis research. 2004;79(2):209–18. doi: 10.1023/B:PRES.0000015391.99477.0d 16228395

[45] Desotgiu R, Cascio C, Pollastrini M, Gerosa G, Marzuoli R, Bussotti F. Short and long term photosynthetic adjustments in sun and shade leaves of Fagus sylvatica L., investigated by fluorescence transient (FT) analysis. Plant Biosystems-An International Journal Dealing with all Aspects of Plant Biology. 2012;146(sup1):206–16. 10.1080/11263504.2012.705350.

[46] Cascio M, Gauson L, Stevenson L, Ross R, Pertwee R, editors. Cannabigerol behaves as a potent alpha-2-adrenoceptor partial agonist. Symposium on the Cannabinoids Burlington, Vermont, International Cannabinoid Research Society; 2009.

[47] Tsimilli-Michael M, Strasser RJ. In vivo assessment of stress impact on plant’s vitality: applications in detecting and evaluating the beneficial role of mycorrhization on host plants. mycorrhiza: Springer; 2008. p. 679–703.

[48] Živčák M, Olšovská K, Slamka P, Galambošová J, Rataj V, Shao H, et al. Application of chlorophyll fluorescence performance indices to assess the wheat photosynthetic functions influenced by nitrogen deficiency. Plant, Soil Environment. 2015;60(5):210–5.

[49] Mehta P, Jajoo A, Mathur S, Bharti S. Chlorophyll a fluorescence study revealing effects of high salt stress on Photosystem II in wheat leaves. Plant Physiology biochemistry. 2010;48(1):16–20. doi: 10.1016/j.plaphy.2009.10.006 19932973

[50] Murkowski A. Oddzialywanie czynnikow stresowych na luminescencje chlorofilu w aparacie fotosyntetycznym roslin uprawnych. Acta Agrophysica. 2002;61:1–158.

[51] Krause G, Weis E. Chlorophyll fluorescence and photosynthesis: the basics. Annual review of plant biology. 1991;42(1):313–49. 10.1146/annurev.pp.42.060191.001525.

[52] Redondo-Gómez S, Mateos-Naranjo E, Davy AJ, Fernández-Muñoz F, Castellanos EM, Luque T, et al. Growth and photosynthetic responses to salinity of the salt-marsh shrub Atriplex portulacoides. Annals of Botany. 2007;100(3):555–63. doi: 10.1093/aob/mcm119 17684026 PMC2533612

[53] Force L, Critchley C, Van Rensen JJ. New fluorescence parameters for monitoring photosynthesis in plants. Photosynthesis research. 2003;78(1):17–33. doi: 10.1023/A:1026012116709 16245061

[54] Kalaji HM, Bosa K, Kościelniak J, Żuk-Gołaszewska K. Effects of salt stress on photosystem II efficiency and CO2 assimilation of two Syrian barley landraces. Environmental Experimental Botany. 2011;73:64–72. 10.1016/j.envexpbot.2010.10.009.

[55] Kalaji H, Rastogi A, Živčák M, Brestic M, Daszkowska-Golec A, Sitko K, et al. Prompt chlorophyll fluorescence as a tool for crop phenotyping: an example of barley landraces exposed to various abiotic stress factors. Photosynthetica. 2018;56(3):953–61. 10.1007/s11099-018-0766-z.

[56] Lotfi R, Kalaji H, Valizadeh G, Behrozyar EK, Hemati A, Gharavi-Kochebagh P, et al. Effects of humic acid on photosynthetic efficiency of rapeseed plants growing under different watering conditions. Photosynthetica. 2018;56(3):962–70. 10.1007/s11099-017-0745-9.

[57] Li F, Vallabhaneni R, Yu J, Rocheford T, Wurtzel ET. The maize phytoene synthase gene family: overlapping roles for carotenogenesis in endosperm, photomorphogenesis, and thermal stress tolerance. Plant physiology. 2008;147(3):1334–46. doi: 10.1104/pp.108.122119 18508954 PMC2442542

[58] Strasser BJ. Donor side capacity of photosystem II probed by chlorophyll a fluorescence transients. Photosynthesis Research. 1997;52(2):147–55. 10.1023/A:1005896029778.

[59] Appenroth K-J, Stöckel J, Srivastava A, Strasser R. Multiple effects of chromate on the photosynthetic apparatus of Spirodela polyrhiza as probed by OJIP chlorophyll a fluorescence measurements. Environmental Pollution. 2001;115(1):49–64. doi: 10.1016/s0269-7491(01)00091-4 11586773

[60] Van Heerden PD, Tsimilli‐Michael M, Krüger GH, Strasser RJ. Dark chilling effects on soybean genotypes during vegetative development: parallel studies of CO2 assimilation, chlorophyll a fluorescence kinetics O‐J‐I‐P and nitrogen fixation. Physiologia Plantarum. 2003;117(4):476–91. doi: 10.1034/j.1399-3054.2003.00056.x 12675738

[61] Abdelaal KAA, Mazrou YSA, Hafez YM. Silicon foliar application mitigates salt stress in sweet pepper plants by enhancing water status, photosynthesis, antioxidant enzyme activity and fruit yield. Plants. 2020;9(6):733. doi: 10.3390/plants9060733 32532054 PMC7356007

[62] Li H, Zhu Y, Hu Y, Han W, Gong H. Beneficial effects of silicon in alleviating salinity stress of tomato seedlings grown under sand culture. Acta physiologiae plantarum. 2015;37(4):1–9. doi: 10.1007/s11738-015-1818-7

[63] Singh P, Kumar V, Sharma J, Saini S, Sharma P, Kumar S, et al. Silicon Supplementation Alleviates the Salinity Stress in Wheat Plants by Enhancing the Plant Water Status, Photosynthetic Pigments, Proline Content and Antioxidant Enzyme Activities. Plants. 2022;11(19):2525. 10.3390/plants11192525. 36235391 PMC9572231

[64] Knox JP, Dodge AD. Singlet oxygen and plants. Phytochemistry. 1985;24(5):889–96. 10.1016/S0031-9422(00)83147-7.

[65] Hamada AM. Effect of NaCl, water stress or both on gas exchange and growth of wheat. Biologia plantarum. 1996;38(3):405–12. 10.1007/BF02896671.

[66] Li P-H, Wang Z-L, Zhang H, Wang B-S. Cloning and expression analysis of the B subunit of VH^+-ATPase in leaves of halophyte Suaeda salsa under salt stress. Journal of Integrative Plant Biology. 2004;46(1):93–9.

[67] Shah SH, Houborg R, McCabe MF. Response of chlorophyll, carotenoid and SPAD-502 measurement to salinity and nutrient stress in wheat (Triticum aestivum L.). Agronomy. 2017;7(3):61. 10.3390/agronomy7030061.

[68] Pastuszak J, Dziurka M, Hornyák M, Szczerba A, Kopeć P, Płażek A. Physiological and Biochemical Parameters of Salinity Resistance of Three Durum Wheat Genotypes. International Journal of Molecular Sciences. 2022;23(15):8397. doi: 10.3390/ijms23158397 35955532 PMC9369059

[69] Isayenkov S, Genetics. Physiological and molecular aspects of salt stress in plants. Cytology. 2012;46(5):302–18. 10.3103/S0095452712050040.

[70] Sarwar G, Ashraf MY, Naeem M. Genetic variability of some primitive bread wheat varieties to salt tolerance. Pakistan Journal of Botany. 2004;35(5; SPI):771–8.

[71] Ashraf M, Foolad MR. Pre‐sowing seed treatment—A shotgun approach to improve germination, plant growth, and crop yield under saline and non‐saline conditions. Advances in agronomy. 2005;88:223–71. 10.1016/S0065-2113(05)88006-X.

[72] Ashraf MY, Sarwar G. Salt tolerance potential in some members of Brassicaceae physiological studies on water relations and mineral contents. Prospects for saline Agriculture: Springer; 2002. p. 237–45.

[73] Yasar F, Uzal O, Tufenkci S, Yildiz K. Ion accumulation in different organs of green bean genotypes grown under salt stress. Plant Soil Environment. 2006;52(10):476. 10.17221/3469-pse.

[74] Gorham J. Salt tolerance in the Triticeae: K/Na discrimination in synthetic hexaploid wheats. Journal of Experimental Botany. 1990;41(5):623–7. 10.1093/jxb/41.5.623.

[75] Kim HU. Lipid metabolism in plants. Plants. 2020;9(7):871. doi: 10.3390/plants9070871 32660049 PMC7411677

[76] van Meer G, Voelker DR, Feigenson GW. Membrane lipids: where they are and how they behave. Nature reviews. Molecular cell biology Nature Reviews Molecular Cell Biology. 2008;9(2):112–24. 10.1038/nrm2330. 18216768 PMC2642958

[77] Morales M, Munné-Bosch S. Malondialdehyde: Facts and Artifacts. Plant Physiology. 2019;180(3):1246–50. doi: 10.1104/pp.19.00405 31253746 PMC6752910

[78] Sadak MS. Physiological role of trehalose on enhancing salinity tolerance of wheat plant. Bulletin of the National Research Centre. 2019;43(1):1–10. 10.1186/s42269-019-0098-6.

[79] Zheng Y, Brash AR. On the role of molecular oxygen in lipoxygenase activation: comparison and contrast of epidermal lipoxygenase-3 with soybean lipoxygenase-1. Journal of Biological Chemistry. 2010;285(51):39876–87. doi: 10.1074/jbc.M110.180794 20923767 PMC3000969

[80] Ayala A, Muñoz MF, Argüelles S. Lipid peroxidation: production, metabolism, and signaling mechanisms of malondialdehyde and 4-hydroxy-2-nonenal. Oxidative medicine cellular longevity. 2014;2014(360438):1–31. doi: 10.1155/2014/360438 24999379 PMC4066722

[81] Baker CJ, Orlandi EW. Active oxygen in plant pathogenesis. Annual review of phytopathology. 1995;33(1):299–321. doi: 10.1146/annurev.py.33.090195.001503 18999963

[82] Bannenberg G, Martínez M, Hamberg M, Castresana C. Diversity of the enzymatic activity in the lipoxygenase gene family of Arabidopsis thaliana. Lipids. 2009;44(2):85. doi: 10.1007/s11745-008-3245-7 18949503

[83] Gill SS, Tuteja N. Reactive oxygen species and antioxidant machinery in abiotic stress tolerance in crop plants. Plant physiology biochemistry. 2010;48(12):909–30. doi: 10.1016/j.plaphy.2010.08.016 20870416

[84] Thapa B, Shrestha A. Protein Metabolism in Plants to Survive against Abiotic Stress: IntechOpen; 2022.

[85] Ashraf M, Harris PJ. Photosynthesis under stressful environments: an overview. Photosynthetica. 2013;51(2):163–90. 10.1007/s11099-013-0021-6.

[86] Liang X, Zhang L, Natarajan SK, Becker DF. Proline mechanisms of stress survival. Antioxidants & redox signaling. 2013;19(9):998–1011. doi: 10.1089/ars.2012.5074 23581681 PMC3763223

[87] Ashraf M. Some important physiological selection criteria for salt tolerance in plants. Flora-Morphology, Distribution, Functional Ecology of Plants. 2004;199(5):361–76. 10.1078/0367-2530-00165.

[88] Wimmer MA, Mühling KH, Läuchli A, Brown PH, Goldbach HE. The interaction between salinity and boron toxicity affects the subcellular distribution of ions and proteins in wheat leaves. Plant, Cell & Environment. 2003;26(8):1267–74. 10.1046/j.0016-8025.2003.01051.x.

[89] Parvaiz A, Satyawati S. Salt stress and phyto-biochemical responses of plants-a review. Plant Soil Environment. 2008;54(3):89.

[90] Parida AK, Das AB, Mittra B, Mohanty P. Salt-stress induced alterations in protein profile and protease activity in the mangrove Bruguiera parviflora. Zeitschrift für Naturforschung C. 2004;59(5–6):408–14. doi: 10.1515/znc-2004-5-622 18998411

[91] Sairam RK, Rao KV, Srivastava GC. Differential response of wheat genotypes to long term salinity stress in relation to oxidative stress, antioxidant activity and osmolyte concentration. Plant science. 2002;163(5):1037–46. 10.1016/S0168-9452(02)00278-9.

[92] Chen Z, Cuin TA, Zhou M, Twomey A, Naidu BP, Shabala S. Compatible solute accumulation and stress-mitigating effects in barley genotypes contrasting in their salt tolerance. Journal of experimental botany. 2007;58(15–16):4245–55. doi: 10.1093/jxb/erm284 18182428

[93] Chen H, Jiang J-G. Osmotic adjustment and plant adaptation to environmental changes related to drought and salinity. Environmental Reviews. 2010;18(NA):309–19. 10.1139/A10-014.

[94] Klähn S, Hagemann M. Compatible solute biosynthesis in cyanobacteria. Environmental microbiology. 2011;13(3):551–62. doi: 10.1111/j.1462-2920.2010.02366.x 21054739

[95] Siddique A, Kandpal G, Kumar P. Proline accumulation and its defensive role under Diverse Stress condition in Plants: An Overview. Journal of Pure Applied Microbiology. 2018;12(3):1655–9. 10.22207/JPAM.12.3.73.

[96] Hayat S, Hayat Q, Alyemeni MN, Wani AS, Pichtel J, Ahmad A. Role of proline under changing environments: a review. Plant signaling behavior. 2012;7(11):1456–66. doi: 10.4161/psb.21949 22951402 PMC3548871

[97] Kuznetsov VV, Shevyakova N. Proline under stress: biological role, metabolism, and regulation. Russian Journal of plant physiology. 1999;46(2):274–87.

[98] Mwadzingeni L, Shimelis H, Tesfay S, Tsilo TJ. Screening of bread wheat genotypes for drought tolerance using phenotypic and proline analyses. Frontiers in plant science. 2016;7:1276. doi: 10.3389/fpls.2016.01276 27610116 PMC4997044

[99] Sharma P, Jha AB, Dubey RS, Pessarakli M. Reactive oxygen species, oxidative damage, and antioxidative defense mechanism in plants under stressful conditions. Journal of botany. 2012;2012. 10.1155/2012/217037.

